# Urinary Tract Infection among Renal Transplant Recipients in Yemen

**DOI:** 10.1371/journal.pone.0144266

**Published:** 2015-12-10

**Authors:** Adnan S. Gondos, Khaled A. Al-Moyed, Abdul Baki A. Al-Robasi, Hassan A. Al-Shamahy, Naelah A. Alyousefi

**Affiliations:** 1 Department of Medical Microbiology, Faculty of Medicine and Health Sciences, Sana'a University, Sana’a, Yemen; 2 Department of Medicine, Faculty of Medicine, University of Malaya, 50603, Kuala Lumpur, Malaysia; Emory University, UNITED STATES

## Abstract

Urinary tract infection (UTI) is the most common complication following kidney transplantation (KT), which could result in losing the graft. This study aims to identify the prevalence of bacterial UTI among KT recipients in Yemen and to determine the predisposing factors associated with post renal transplantation UTI. A cross sectional study included of 150 patients, who underwent KT was conducted between June 2010 and January 2011. A Morning mid-stream urine specimen was collected for culture and antibiotic susceptibility test from each recipient. Bacterial UTI was found in 50 patients (33.3%). The prevalence among females 40.3% was higher than males 29%. The UTI was higher in the age group between 41–50 years with a percentage of 28% and this result was statistically significant. Predisposing factors as diabetes mellitus, vesicoureteral reflux, neurogenic bladder and polycystic kidney showed significant association. High relative risks were found for polycystic kidney = 13.5 and neurogenic bladder = 13.5. The most prevalent bacteria to cause UTI was *Escherichia coli* represent 44%, followed by *Staphylococcus saprophyticus* 34%. Amikacin was the most effective antibiotic against gram-negative isolates while Ciprofloxacin was the most effective antibiotic against *Staphylococcus saprophyticus*. In conclusion, there is high prevalence of bacterial UTI among KT recipients in Yemen. Diabetes mellitus, vesicoureteral reflux, neurogenic bladder, polycystic kidney and calculi were the main predisposing factors.

## Introduction

Urinary tract infection (UTI) is the most common infection in kidney transplant recipients and is considered a potential risk factor for worse kidney transplantation outcomes [1]. The allograft injury is the main concern caused by bacterial infection. Also acute rejection and calcineurin inhibitor toxicity could be consequences of this infection. According to Veroux *et al* [2], the prevalence of UTI reached up to a 60% during the first year post-transplantation. Other studies reported a prevalence of 45% to 85% respectively [3–5]. Immunosuppressive drugs play a major role for this high prevalence among KT recipients beside the chronic kidney malfunction [2].

There are several risk factors associated with UTI among KT patients, including female gender, old age, diabetes mellitus, immunosuppression, history of vesicoureteral reflux, history of polycystic kidney disease and other risk factors related to the graft and the operation [6]. According to published studies, UTIs were found to be more frequent in patients who was received deceased grafts compared with live grafts and female patients were more susceptible in general [6, 7]. Moreover, a five year follow up recorded at least one episode of UTI among kidney recipients and acute pyelonephritis represented an independent risk factor associated with declining renal function [8]. These findings make following up the patients after kidney transplantation more important due to the fact that UTI could be associated with the early onset of chronic rejection leading to reduced transplant survival which is the main concern for patients. Thus, the prevention of urinary tract infection, or the early diagnosis and accurate treatment of urinary tract infection is critical in renal transplant recipients [9].

The most common cause of UTI is bacteria [10]. Fungi and viruses can also cause UTI among renal transplant recipients, but infections caused by these organisms are less common than those caused by bacteria [11]. The dominant isolated bacteria is *Escherichia coli* (31.5%) followed by *Candida albicans* (21.0%) and *Enterococcus spp* (10.5%) [7]. A long follow-up study revealed that UTI infections were mainly caused by *Escherichia coli* (28.4%), *Pseudomonas aeruginosa* (14.9%) and *Enterobacter cloacae* among kidney transplant recipients [8].

Renal failure is a serious cause of mortality in Yemen. The incidence of end-stage renal disease (ESRD) in Yemen is 120 cases per million per year, which is comparable to the reported incidence in other countries of the same region [12, 13]. Other early data recorded that the incidence of (ESRD) was 385 cases per million per year in Sana’a city the capital of Yemen [14]. According to this, if the incidence is extrapolated over the whole country 7000 new patients with (ESRD) are expected every year [14]. According to Al-Rohani [15] there are about 568 patients with end-stage renal failure (ESRD) who receive chronic haemodialysis in seven centres in Yemen. First kidney transplantation was done in 1998 the early data on allograft was published in 2002 which recorded the first renal transplantation for two cases at Al-Thawra Modern General Hospital, Sana'a [16]. In Yemen, there is only one published data regarding kidney transplantation, where 31 patients followed up between May 1998 and June 2006, (21 male and 10 female), all of whome received a renal allograft from live related donors at the Urology and Nephrology Centre at Al-Thawra Modern General Hospital Sana'a, Republic of Yemen [15]. Unfortunately, this published data did not cover the UTI burden after the surgery. The present study is the first to study the incidence of UTI among kidney transplant recipients in Yemen.

## Materials and Methods

This cross-sectional study which started in June 2010 and ended in January 2011 was conducted in Sana’a city on a total of 150 kidney transplant recipients. The age of patients ranged from 17 to 71 years old, the mean age for males was 35.6 years and for females was 34.4 years. Most of them had their KT outside the country 117 (78%) while only 33 (22.2%) underwent the surgery in Sana’a, Yemen. Patients came from different areas for follow-up at the Nephrology & Urology center at Al-Thawrah hospital and at Dr. Nageeb Abu Isba’s kidney centre in Sana'a city. This study including the questionnaire and the consent form was approved by the Ethic Committee at Faculty of Medicine and Health Sciences, Sana’a University on their 6^th^ meeting 2009–2010 (FOMHS-4-4-2010). All the patients were invited voluntarily after a clear explanation about the study objectives. Written consent forms were obtained from the all volunteers. Written consent forms for patients less than 18 years were obtained from their parents. Permission was also obtained from the manager of Althwra hospital and Dr Najeeb Abu Isba the consultant in the kidney center. None of the transplant donors were from a vulnerable population and all donors or next of kin provided written informed consent that was freely given. All the patient’s records were anonymized by giving a number to each sample and questionnaire before the analysis. All sociodemographic and clinical history of the patient were recorded in the pre-designed questionnaire. Data analyses was done using SPSS (Statistical Package for Social Sciences) computer program (Version 15.0). Statistical analyses for significant association have been done using the probability value (*p*) with (confidence interval (CI) of 95% and *p* value less than 0.05) and relative risk (RR) **(χ** ^**2**^≥3.84).

### Sample collection and microbial culturing

A morning clean-catch mid stream urine (MSU) specimens was collected in a sterile, dry and leak-proof container from each patient. Samples were labeled and subjected immediately to macroscopic and microscopic examination. Routine urine microscopic examination and one time bacterial culture have been done for all the patients. One μl of the specimen was inoculated onto blood agar plate and MacConkey agar plate (HiMedia, India). These were incubated aerobically at 35–37°C for 24–48 hours. Sub culturing the bacterial isolates on suitable biochemical media for confirmation. Antibiotic susceptibility testing was performed for every positive culture. Cultures were considered positive if 100 CFUs or more of pure growth were obtained on the plates according to the Infectious Diseases Society of America (IDSA) guidelines [17]. All the cultures media were prepared and inoculated according to the manufacturer's instructions.

Identification of bacteria in positive culture was based on standard methods, these included Grams staining of smears and biochemical tests relevant to the isolate of species. The biochemical tests include Kligler Iron Agar (KIA), Sulfide, Indole, Motility (SIM), citrate and methyl red. Also the routine bench tests such as catalase, oxidase and coagulase were done. Mueller-Hinton agar was used to do the susceptibility testing for the isolated bacteria. An antibiogram relevant to UTIs which represented the different groups of antibiotics (for both Gram-negative and Gram-positive bacteria) was used in susceptibility testing. The antibiotic discs, beside all the Commercial kits of biochemical tests and culture media were manufactured by HiMedia, India.

## Result

The prevalence of renal transplant recipients according to the governorates in Yemen, Sana’a showed that has the highest number of patients while the lowest number was found in Lahj and Al-Mahweet governorates equally. The majority of the renal transplant recipients was in the age group of 21–30 years old with a percentage of 36.7% and the lowest age group was 17–20 years old age with a percentage of 7.3%. Males represented 62% and females 38% respectively. Bacterial UTI was diagnosed in 50 (33.3%) of the total studied recipients. Female showed higher UTI infection than male but without statistical difference. Female and male percentage was 40.3% and 29% respectively (Table 1). The older age group (41–50) years showed a significant association with bacterial UTI (28%) (χ2 = 13.24 and *p* < 0.05). The younger group had a much lower risk of UTI only 2%. The live-related group represented 70% but only 34.3% had UTI. The live-unrelated group represented 30% and UTI prevalence was 31.1%. So, there was no significant difference among these groups with UTI.

**Table 1:** Distribution of bacterial UTI among the total renal transplant recipients according to Gender, Place of the transplantation and the type of donation in Yemen, 2011.

Variables	With UTI		Without UTI		Total		χ^2^	*p*
	n = 50		n = 100		n = 150			
	No	%	No	%	No	%		
Gender								
Male (n = 93)	27	29.0	66	71	93	62		
Female (n = 57)	23	40.3	34	59.7	57	38.0	2.037	0.153
Age 5 groups 17–20	1	2	10	10	11	7.3		
21–30	12	24	43	43	55	36.7		
31–40	13	26	23	23	36	24	13.24	**0.010**
41–50	14	28	17	17	31	20.6		
51–70	10	20	7	7	17	11.4		
Type of donation								
Live-related (n = 105)	36	34.3	69	66	105	70		
Live unrelated (n = 45)	14	31.1	31	69	45	30	1.543	0.819
Place of the transplantation								
Local (n = 33)	9	27.3	24	73	33	22		
Abroad(n = 117)	41	35.0	76	65	117	78	1.257	0.533

**χ** ^**2**^ Chi-square = ≥ 3.84 **(significant)**.

***P*** Probability value = < 0.05 **(significant)**.

The symptoms showed a high prevalence of burning sensation 78% among UTI and all clinical pictures showed high statistical significance comparing with non-UTI infection, while the highest relative risk was shown among patients who suffered from fever (Table 2). Studying the predisposing factors revealed that diabetes mellitus was the most prevalent (46%). The following factors gave a significant association with UTI: Diabetes Mellitus, Vesicoureteral Reflux, Neurogenic bladder and Polycystic Kidney the *p*<0.05. The relative risk of getting UTI was high for polycystic Kidney and neurogenic bladder (Table 2).

**Table 2:** Distribution of bacterial UTI among total renal transplant recipients according to the symptoms and predisposing factors in Yemen, 2011

Variable	With UTI		Without UTI		RR	CI	χ^2^	*P*
	(n = 50)		(n = 100)					
	No	%	No	%				
Symptoms								
Burning sensation	39	78	7	7	47.104	17.007–130.462	79.029	**<0.0001**
Frequency of urination	23	46	7	7	11.317	4.384–29.216	31.688	**<0.0001**
Urgency	24	48	10	10	8.308	3.526–19.576	27.459	**<0.0001**
Flank pain	26	52	16	16	5.688	2.632–12.290	21.429	**<0.0001**
Urethral pain	25	50	10	10	9.000	3.821–21.201	29.184	**<0.0001**
Fever	27	54	7	7	15.596	6.042–40.261	42.007	**<0.0001**
Dysuria	18	36	8	8	6.469	2.565–16.311	20.481	**<0.0001**
The predisposing Factor								
Diabetes Mellitus	23	46	26	26	2.425	1.188–4.948	6.062	**0.014**
Vesicoureteral Reflux	7	14	4	4	3.907	1.086–14.053	4.905	**0.027**
Neurogenic bladder	6	12	1	1	13.500	1.578–115.50	9.066	**0.003**
Polycystic Kidney	6	12	4	4	13.500	1.578–115.50	9.066	**0.003**
Caculi	6	12	4	4	3.327	0.879–12.18	3.429	0.064

**RR** Relative risk = > 1 **(at risk).**

**CI** Confidence intervals.

**χ** ^**2**^Chi-square = ≥ 3.84 **(significant).**

***p***Probability value = < 0.05 **(significant**).

The distribution of the main causes of kidney failure according to gender showed that the most prevalent cause of renal failure in this study was hypertension, males (51.7%) and females (42%). There was statistical significance difference between male and female for calculi and unknown cause with values of χ^2^ = 6.163, *p*<0.05 and χ^2^ = 8.574, *p*< 0.05 respectively. The relative risk of getting UTI among males with calculi and diabetes mellitus was high; both relative risks were nearly 4 times more than the other causes (Table 3).

**Table 3:** Distribution of the main causes renal failure according to gender in Yemen, 2011.

Main causes	Male (n = 93)		Female (n = 57)		Total (n = 150)		RR	CI	χ^2^	*P*
	No	%	No	%	No	%				
Hypertension	48	51.7	24	42	72	48	1.467	0.754–2.852	1.280	0.258
Calculi	21	22.6	4	7	25	16.7	3.865	1.253–11.922	6.163	0.013
Pyelo-nephritis.	40	43	20	35	60	40	1.396	0.706–2.760	0.924	0.336
Vesicoureteral reflux	14	15	8	14	22	14.7	1.085	0.424–2.776	0.029	0.864
Unknown cause*	17	18.3	23	40	40	26.7	0.331	0.157–0.697	8.574	0.003
Diabetes mellitus	11	11.8	2	3.5	13	8.7	3.689	0.787–17.292	3.090	0.079

**RR**Relative risk = > 1 **(at risk)**.

**CI**Confidence intervals.

**χ** ^**2**^Chi-square = ≥ 3.84 **(significant)**.

***p***Probability value = < 0.05 **(significant)**.

Unknown cause* Patients did not know their renal failure cause.

*Escherichia coli* was the most common bacterial isolated with a percentage of 44% and the least common isolated was *Pseudomonas aeruginosa* 4% (Table 4).

**Table 4:** Prevalence of the 50 bacterial isolates from renal transplant recipients in Yemen, 2011.

Bacteria	No	%
*E coli*	22	44%
*Staph*. *Saprophyticus*	17	34%
*Eenterobacterspp*	6	12%
*Klebsiellaspp*	3	6%
*Psuedomonasaeruginosa*	2	4%
Total	50	100%

### Antibiotic suitability result according to bacteria species (*in vitro*)

***Escherichia coli***: The most effective antibiotic was Amikacin 82% effectiveness, followed by Gentamycin 73%. Ampicillin was the least effective antibiotic, with a percentage of 9%.

***Staph*. *Saprohpyticus***: Ciprofloxacin and Doxycycline were the most effective antibiotics with a percentage of 64% for each. Naldixic acid was the least effective 6%.

***Enterobacter spp***: Amikacin was the most effective antibiotic 67%, followed by Ciprofloxacin 66%. Naldixic acid gave less effect with a percentage of 17%.

***klebsiella spp***: Amikacin was the most effective antibiotic, with a percentage of 100%, followed by ciprofloxacin with a percentage of 66%. Naldixic acid was the least effective antibiotic with a percentage of 17%.

***Pseudomonas aeruginosa*:** Amikacin, Ofloxacin, Ciprofloxacin and Norfloxacin were the most effective antibiotics, with a percentage of 100% for each. Naldixic acid, Doxycycline and Cefuroxime were the least effective antibiotics, *in vitro*, with a percentage of 0% for each.

## Discussion

In the present study, the prevalence of bacterial UTI among renal transplant recipients is high in Yemen (33.3%). This result is nearly identical to that reported by Shirazi *et al*. [18] in Iran with 33%. This is not surprising, since many publications showed these high incidences among the kidney transplant recipients in Brazil [19], among African American [3], Mexico [20] and in Libya by Elkehili *et al*. [21]. The high prevalence of UTI after kidney transplantation could be because of the immunosuppressive drugs with the high dosage that could have been taken by patients [22]. This result based on one sample culturing at random time after KT while post transplantation prevalence after follow up for one year showed higher prevalence reached to 45% to 85% respectively [3–5] and this consider as a limitation in the current study. Also no comparison has been preformed between UTI and immunosuppressive therapy that given to KT patients…

Females are more affected than males (40.3% versus 29%) but this result showed no statistical significance with UTI as most of the patients are male. A similar finding conducted by Shirazi *et al*. [18] in Iran showed the infection in females was 37% versus 32% in males with no observation of a significant variance between both sexes. Otherwise the risk of being female had a significant association with UTI in several studies [6, 21–24]. According to the recently published data from Yemen on patients who attended hospitals with UTI 53.3% of them were females [25]. In a retrospective cohort study of 28,942 Medicare primary renal transplant recipients, the United States Renal Data System (USRDS) found that the cumulative incidence of UTI at the 3^rd^ year was 60% for women and 47% for men [26]. Most findings which were performed on UTI show that infection was more prevalent among females than males due to different factors such as pathogenicity and host factors. Recently published data from Turkey found the risks of getting UTI depended on the rate and duration of ureteral catheterization [24].

UTI also increases among older patients (41–50 years old) (28%) with significant association comparing with younger age groups. This finding is compatible with a study by Chuang *et al*. 2005 where patients 65 years or older developed post-transplant UTI with 55% compared to younger patients. The earliest review on asymptomatic bacteriurea reported an increase of UTI to be 50% for elderly women and 15%–40% for elderly men [26]. In the same way, Nicolle [27] reported that prevalence increases with advancing age to reach about (6%) at 60 years of age and to 15% in men over 75 years of age. In addition to that, a recently published paper by Alkhyat and Maqtari [25] on patients attending hospitals recorded a high prevalence of UTI in the age group >40 years old as 58.62% in males and 40% `in females. The high prevalence of UTI among the elderly age group in the present study may be attributed to a biological or behavioural predisposition of the host for uropathogenic strains and decreased immunity with advanced age.

All the patients received the kidney from a living donors and no risk of getting UTI has been found whether the kidney donor’s is livd-related or lived unrelated. An exception to this finding is a study in Libya by Elkehili *et al* [21] among renal transplant recipients; which found that those recipients who received the allograft from live-unrelated donors had more UTIs than live-related ones and no statistical significance had been recorded.

The main cause of renal failure among the participants in this study was hypertension followed by calculi, polynephritis, vesicoureteral reflux, unknown cause and the least cause was diabetes mellitus. Hypertension showed high prevalence among men than women 51.7% and 45% respectively. An earlier report from Yemen addressed Malaria, diarrhea and high fever among children with some adults as a reason for renal failure while hypertension was not mentioned as a cause of renal failure [14]. Many reasons can cause renal failure and, a study conducted by John *et al* [28] in Germany, reported that the main primary renal end stage disease was due to glomerular disorders, followed by urinary tract malformation and renal dysplasia and to a lesser extent due to metabolic disorders, polycystic kidney disease and neurogenic bladder. On other hand, Iqbal *et al* [29] reported that the underlying causes for end stage renal failure included calculi disease, glomerulonephritis, hypertensive nephropathy, diabetic nephropathy, adult polycystic kidney disease and unknown causes. In fact hypertension could be associated with renal dysfunction and not to be the main cause of kidney failure. In this respect, an observational study found intensive blood-pressure control had no effect on kidney disease progression [30]. The current finding could reflect the need for more studies to confirm the association of hypertension with kidney failure among Yemenis. In addition to that, we found that calculi and unknown cause showed an association with gender. Hence UTI associated with calculi among males, while unknown cause was associated with female UTI infection. This finding is subscribed to an earlier report where the main cause of Kidney failure among Yemenis receiving KT in Jordan was obstructive uropathy caused by stones [31]. The reason for kidney stones could be attributed to dietary and lifestyle factors which are likely to play an important role in the changing epidemiology of kidney stones. The prevalence of stone disease in the United States showed an increase among males more than females 10.6% -7.1% respectively [32]. Nevertheless, the present study and the Iqpal *et al* [29] study showed different causes of end stage renal disease, but both agreed that a high proportion of recipients did not know the causes of their renal failure.

All the patients in the current study have showed clinical symptoms and burning sensation was the most prevalent among renal transplant recipients with bacterial UTI. The least described was dysuria with a percentage of 78% versus 36%. All these symptoms showed a statistical significance with a high relative risk of contracting UTI. Al-Awadi [33] (unpublished data) listed a burning sensation as the most frequent symptoms related with symptomatic UTI. It was also one of the complications of UTI with oliguria, diarrhoea, hypertension, nausea, vomiting, fever and pain after kidney transplantation according to the recent study by Procópio *et al*. [34]. While in other study no symptoms have been reported by half of the patients, who had febrile illness and the minority of these patients had dysuria and flank pain [29]. In contrast, according to a recent finding asymptomatic UTI among kidney transplantation was the most common manifestation of bacteriuria after renal transplantation [35].

The current study found that diabetes mellitus was the major predisposing factor for UTI among these recipients with a percentage of 46% and the least was neurogenic bladder, vesicoureteral reflux and polycystic kidney with a percentage of 12% for each. All these factors showed a statistical significance; except for calculi, and all of those factors had high relative risk to cause UTI infection. Al-Awadi, (2004) in Yemen reported that half of the studied UTI patients were diabetic. Being a diabetic was a risk factor for UTI among females according to a review by Khanna *et al* [36]. Also glucosuria was relatively high which may indicate a high incidence of diabetic mellitus elevated amongst these recipients after transplantation as concluded by Alangaden *et al*., [3] that was explained by the post-transplant state of hyperglycemia or new-onset diabetes as developed as a primary consequence of the immunosuppression.

The most prevalent bacteria causing UTI in this study is *Escherichia coli* with a percentage of 44%, followed by *Staph*. *saprophyticus* 34%, then *Enterobacter spp* 12%, *Klebsiella* spp 6%, and finally *Pseudomonas aeruginosa* 4%. This result is compatible with an early review paper where *Escherichia coli* was the most frequent aetiology for UTI among kidney transplant patients [37]. Recent published data showed that the most common infectious agents were *Escherichia coli* and *Klebsiella pneumoniae*, for both isolated and recurrent UTI among renal transplant patients [19]. In Saudi Arabia, a study for post-renal transplant UTI found that *Escherichia coli* was the most common pathogen (53.3%) followed by *Pseudomonas aeruginosa* (20%) [38]. Many studies stated that the most common pathogen isolated from renal transplant recipients was *Escherichia coli* [29, 30, 36].

This study showed that Amikacin was the most effective antibiotic, against all Gram negative bacilli, Ciprofloxacin and Doxycycline were the most effective antibiotics, against Gram positive cocci. In contrast, Naldixic acid and Ampicllin were the least effective antibiotics. All patients received prophylactic antibiotics directly after surgery. The low resistance for antibiotic in this study indicated that the infection could happen after the kidney transplantation for enough long periods and not the resulted of using catheters. High resistance had been reported against Amikacin and Ciprofloxacin by Elkehili *et al*. [21] this agreed to some extent with the present result in that the most effective antibiotic was Ciprofloxacin. Senger *et al* [39] described high resistance rates for *Escherichia coli* against ciprofloxacin but this study showed moderate susceptibility against Ciprofloxacin for the same organism. It can be seen from the above mentioned results that the susceptibility to different isolates towards antibiotics in UTI varied from one country to another and from one researcher to another. This depends on the therapy regimen in those countries and the public understanding of antibiotic misuse from one side, beside the use of standard antibiotic susceptibility testing technique by researchers from another side. Prophylaxis against UTI through hygiene practices should be followed by kidney transplant patients especially that they have low immunity

## Conclusion

Currently this is the first study which has been done among patients after kidney transplantation in Yemen and it intended to help in the understanding the causes of UTI among kidney transplant recipients. The UTIs prevalence among Kidney recipients was high and the old age group recipients had high risk of getting UTI. Diabetes mellitus, vesicoureteral reflux, neurogenic bladder, polycystic kidney and calculi was the main predisposing factors, *Escherichia coli* was the dominant causative agents of bacterial UTI among these recipients in Yemen. More studies concerning the predisposing factors are very important to avoid any complications after KT. A long term following up and a comparison of immunosuppressive therapy and UTI a among KT patients should be considered in the future research plane to give accurate picture for UTI after KT among Yemenis. A comparison of immunosuppressive therapy and UTI should be performed.
